# Supplementary material for: Activation of the integrated stress response by inhibitors of its kinases
Source: Nat Commun. 2023 Sep 8;14:5535. doi: 10.1038/s41467-023-40823-8 (PMC10491595; doi:10.1038/s41467-023-40823-8)
Supplement: Supplementary file 1 — Supplementary Information [file 41467_2023_40823_MOESM1_ESM.pdf]

Supplementary Figures

Supplementary Fig. 1

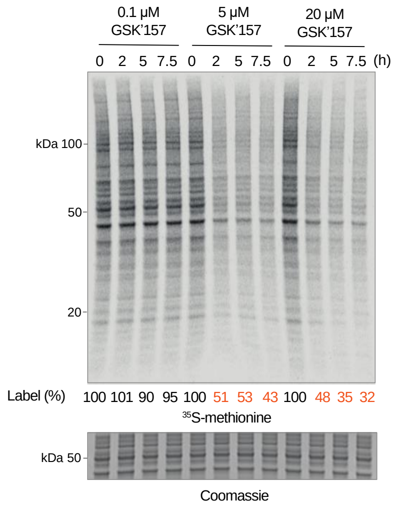

GSK'157 induces persistent translational attenuation in unstressed cells. Newly synthesized proteins labelled for 10 min with ^{35}S-methionine in HeLa cells treated with indicated concentrations of GSK'157 for the indicated times and a Coomassie-stained gel as control. Representative experiment from n=2, biologically independent experiments. Source data are provided as a Source Data file.

Supplementary Fig. 2

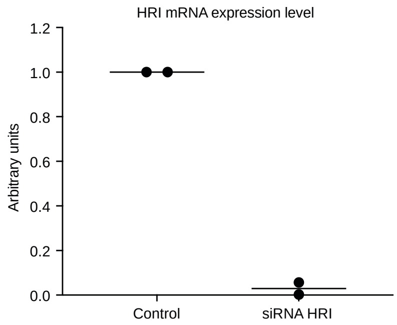

Efficiency of HRI siRNA treatment in HeLa cells. Relative abundance of HRI mRNAs detected by qPCR in lysates from HeLa cells untreated or pre-treated with HRI siRNA for 60 h. Data are mean of n=2, biologically independent experiments. Source data are provided as a Source Data file.

Supplementary Fig. 3

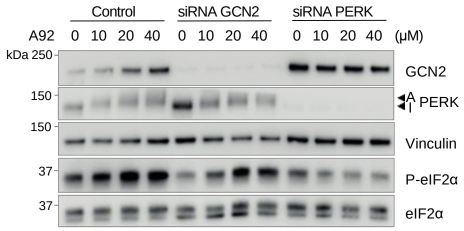

Activation of PERK and eIF2α phosphorylation by A92 are independent of GCN2. Immunoblots of indicated proteins in lysates from HeLa cells untreated or pre-treated for 60 h with indicated siRNA and then subjected to treatment with increasing concentrations of the A92 GCN2 inhibitor for 5 h. Active (A) and Inactive (I) PERK are indicated with arrows. Representative experiment from n=2, biologically independent experiments. Source data are provided as a Source Data file.

Supplementary Fig. 4

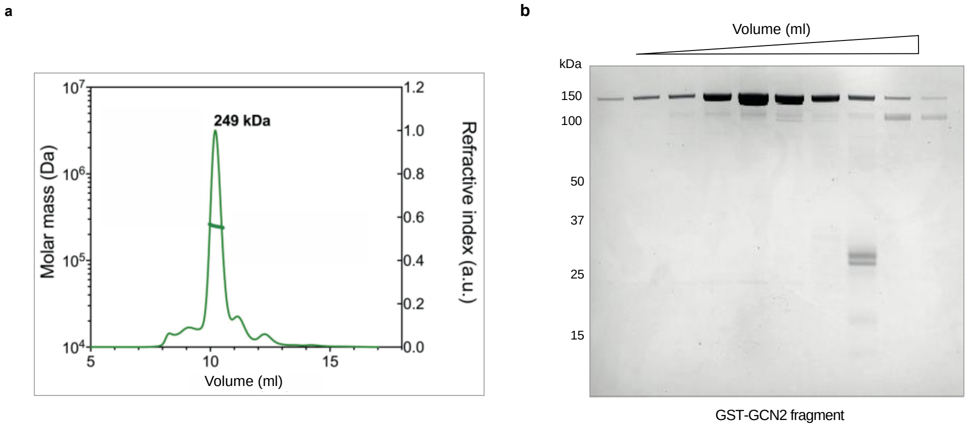

GST-GCN2 fragment is dimeric. a Size-exclusion chromatography–multiangle light scattering (SEC-MALS) profile of in-house recombinant GST-GCN2 fragment (MW ~ 135 kDa). The molecular weight measured by light scattering was 249 kDa consistent with a dimeric state of the recombinant protein. Representative experiment from n=2, biologically independent experiments. **b** Coomassie-stained gels of fractions from panel a, SEC-MALS analysis of GST-GCN2 fragment. Representative experiment from n=2, biologically independent experiments. Source data are provided as a Source Data file.

Supplementary Fig. 5

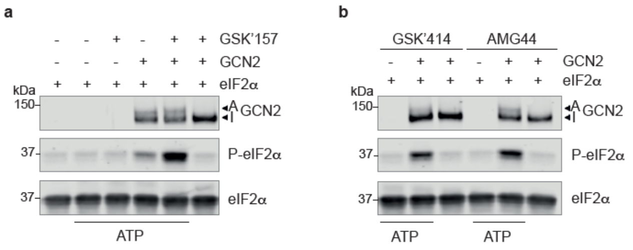

The kinase activating activity of PERK inhibitors GSK'157, GSK'414 and AMG44 depends on GCN2 and ATP in in vitro assays. a Immunoblots of indicated proteins from in vitro kinase reaction carried out with or without 7.5 nM GCN2, with or without 5μM GSK'157, with or without 25 μM ATP and all with 2 μM eIF2α. Reactions were incubated for 20 min at 30 °C. Active (A) and Inactive (I) GCN2 are indicated with arrows. Representative experiment from n=3, biologically independent experiments. **b** Immunoblots of indicated proteins from in vitro kinase reactions as in a, with or without 7.5 nM GCN2, with or without 25 μM ATP and with 5 μM GSK'414 or 5 μM AMG44 as indicated. Active (A) and Inactive (I) GCN2 are indicated with arrows. Representative experiment from n=3, biologically independent experiments. Source data are provided as a Source Data file.

Supplementary Fig. 6

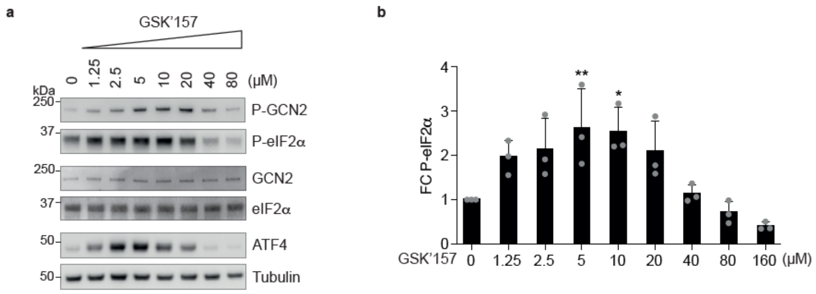

Gaussian activation profile of GCN2 and ISR activation in cells by GSK'157. a Immunoblots of indicated proteins from lysates of HeLa cells treated with indicated concentrations of the PERK inhibitor GSK'157. Representative experiment from n=3, biologically independent experiments. **b** Quantification of P-eIF2α levels normalized to untreated cells. Data are shown as mean ± SD n=3, biologically independent experiments. *$p<0.011$, **$p<0.008$ as determined by one-way ANOVA with Dunnett's multiple comparison test. Source data are provided as a Source Data file.

Supplementary Fig. 7

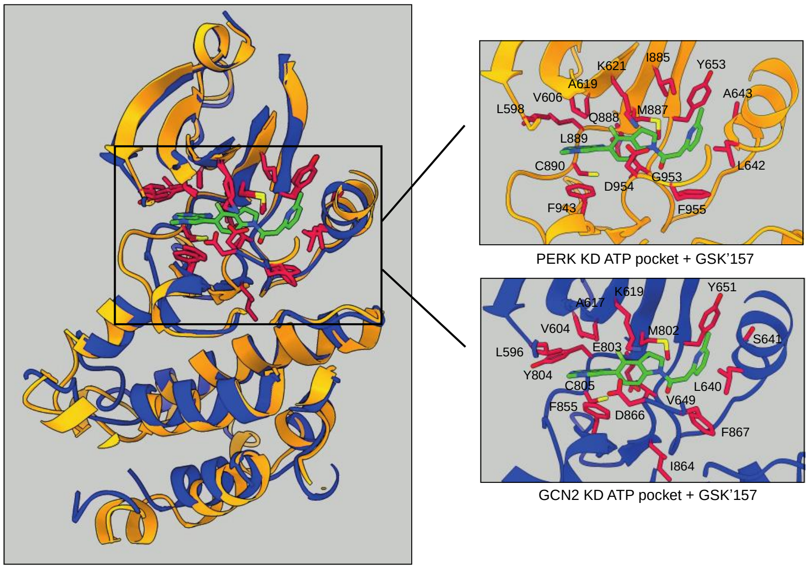

GSK’157 in PERK and GCN2 ATP-binding site. Structure of PERK kinase domain (yellow) with GSK’157 in the ATP-binding site reported in[1] superimposed with the structure of GCN2 kinase domain (blue) from[2] using ChimeraX software. The high homology between the ATP-binding sites of both kinases allows unambiguous fitting of GSK’157 into the ATP-pocket of GCN2 kinase. GSK’157 is highlighted in green with interacting residues in both kinases marked in red.

Supplementary Fig. 8

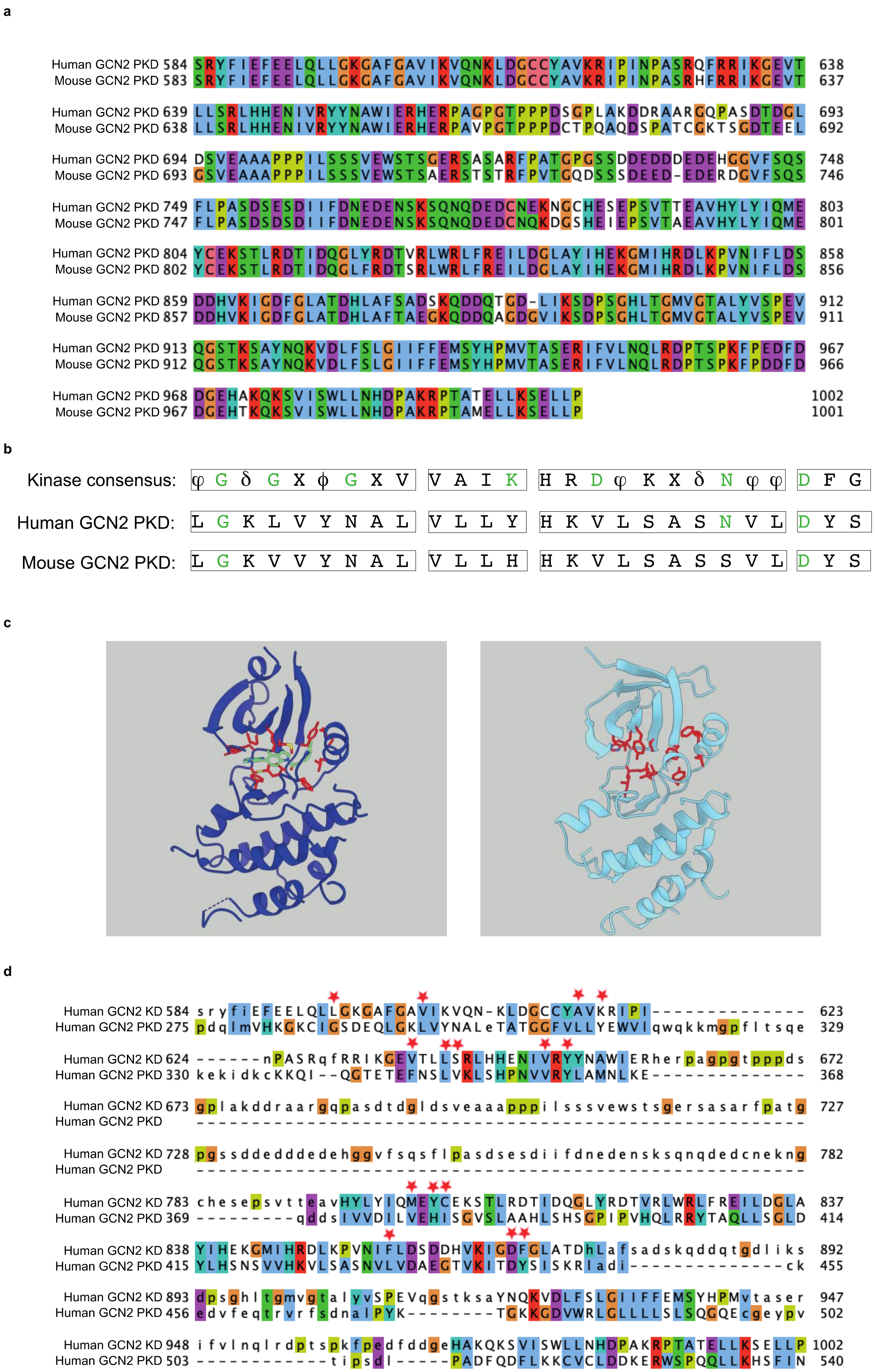

Analyses of the pseudokinase and kinase domains of GCN2. a Sequences of GCN2 pseudokinase domains (PKD) from *H. sapiens* (Q9P2K8) and *M. Musculus* (Q9QZ05) were aligned using Clustal Omega[3] and coloured according to Clustal colouring scheme. Colour intensity indicates sequence conservation. **b** Consensus sequence of the protein kinase catalytic motifs compared with respective residues from the PKD of human GCN2 and mouse GCN2. Residues coloured in green are essential for effective catalytic activity. φ = hydrophobic amino acid, δ = hydrophilic amino acid, ϕ = large hydrophobic amino acid, X = any amino acid[4]. **c** Side by side comparison of GCN2 kinase domain (KD, dark blue) and GCN2 PKD (light blue). GSK'157 docked on GCN2 KD is shown in green. KD residues that are in contact with the GSK'157 and the corresponding residues in GCN2 PKD are shown in red. **d** Structure-based sequence alignment of GCN2 PK and PKD. Residues that make potential contacts with GSK'157 are indicated with a red star.

Supplementary Fig. 9

a

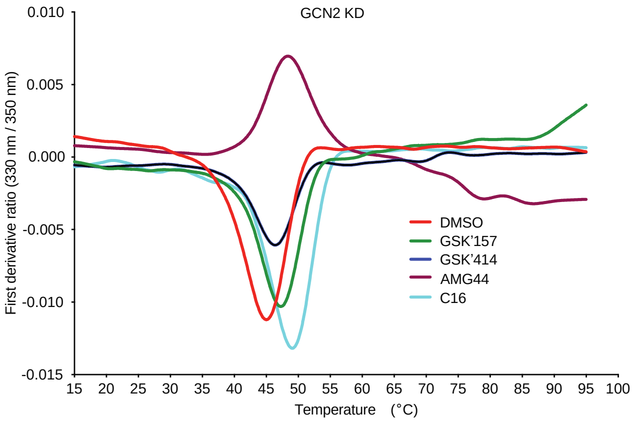

GCN2 KD	Tm +/- SD (°C)
DMSO	44.9 +/- 0.2
GSK'157	47.1 +/- 0.2
GSK'414	46.3 +/- 0.1
AMG44	47.9 +/- 0.4
C16	49.0 +/- 0.1

b

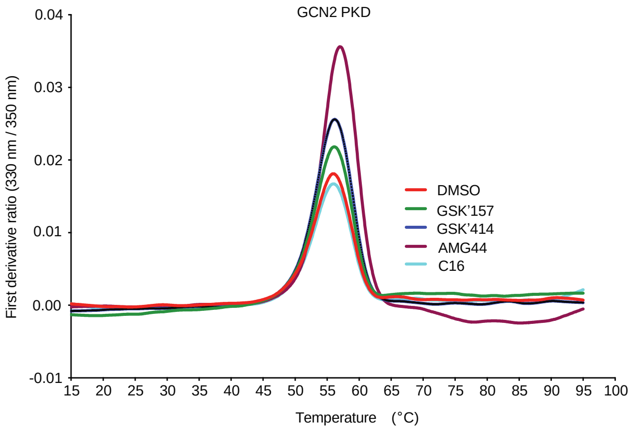

GCN2 PKD	Tm +/- SD (°C)
DMSO	56.0 +/- 0.2
GSK'157	56.1 +/- 0.1
GSK'414	56.2 +/- 0.0
AMG44	57.0 +/- 0.0
C16	56.0 +/- 0.0

c

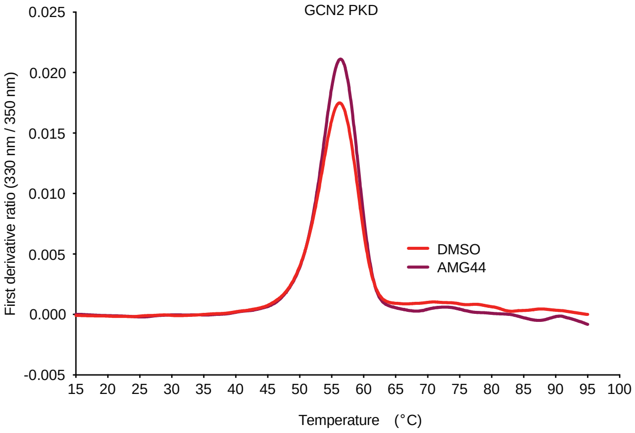

GCN2 PKD	Tm +/- SD (°C)
DMSO	56.1 +/- 0.1
AMG44	56.3 +/- 0.0

Diverse GCN2 activators bind the kinase domain but not the pseudokinase domain of GCN2. **a** Thermal stability of GCN2 kinase domain (KD) in the presence or absence of indicated compounds tested (100 μM). Representative experiment from n=3, biologically independent experiments. Melting temperature of the protein (T_m) shown as mean ± SD. **b** Thermal stability of

GCN2 pseudokinase domain (PKD) in the presence or absence of indicated compounds tested (100 μM). Representative experiment from n=3, biologically independent experiments. Protein T_m shown as mean ± SD. **c** AMG44 yielded a modest T_m shift at 100 μM (**b**), but no shift was observed at 10 μM. Representative experiment from n=3, biologically independent experiments. Protein T_m shown as mean ± SD. Source data are provided as a Source Data file.

Supplementary Fig. 10

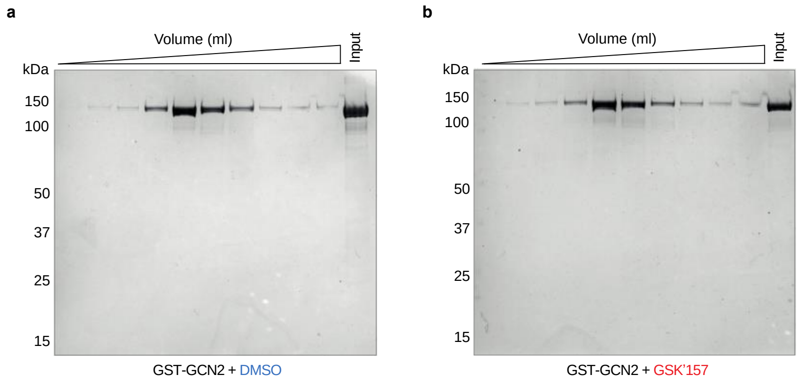

GSK'157 does not influence the dimerization status of GST-GCN2 fragment. a-b Coomassie-stained gels of fractions from SEC-MALS analysis of GST-GCN2 fragment in presence of DMSO or 50 μM GSK'157 shown in Fig. 7a. Representative experiment from n=2, biologically independent experiments. Source data are provided as a Source Data file.

Supplementary Fig. 11

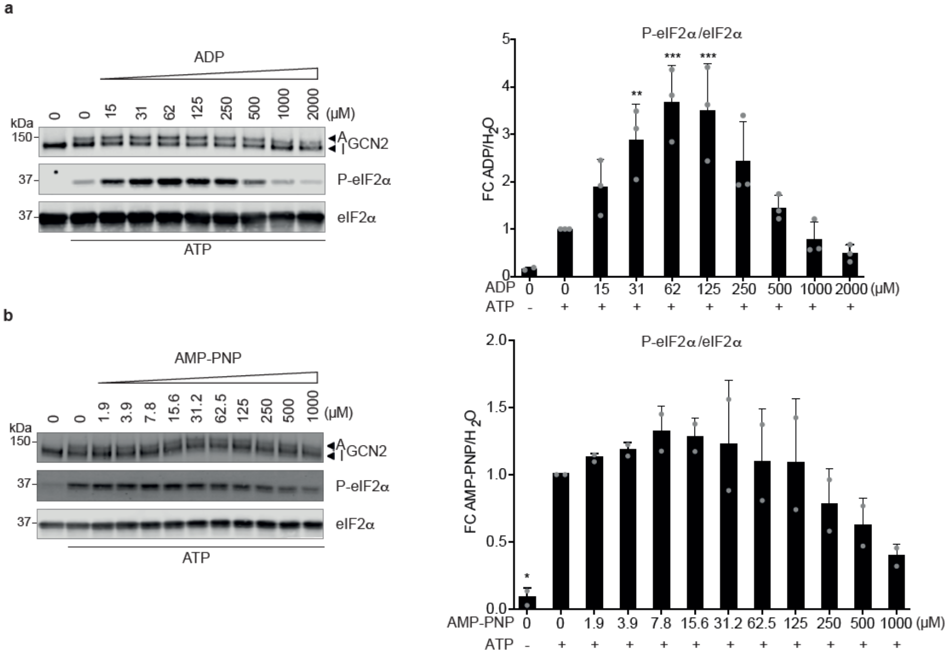

ADP but not AMP-PNP activates GCN2 and induces eIF2α phosphorylation in vitro. a Immunoblots of indicated proteins from in vitro kinase experiments carried out with 7.5 nM GCN2, 6 µM ATP, 2 µM eIF2α and with indicated concentrations of ADP. Reactions were incubated for 20 min at 30°C. Active (A) and Inactive (I) GCN2 are indicated with arrows. Representative experiment from n=3, biologically independent experiments. Ratio of P-eIF2α to eIF2α levels in immunoblots such as shown in the right panel normalised to 6 µM ATP conditions. Data are shown as mean ± SD (n=3, except no ATP conditions n=2), biologically independent experiments. **p<0.01, ***p<0.0006 as determined by one-way ANOVA with Dunnett's multiple comparison test. **b** Immunoblots of indicated proteins from in vitro kinase reaction as in a, carried out with 7.5 nM GCN2, 12 µM ATP, 2 µM eIF2α and with indicated concentrations of AMP-PNP. Active (A) and Inactive (I) GCN2 are indicated with arrows. Representative experiment from n=2, biologically independent experiments. Ratio of P-eIF2α to eIF2α levels in immunoblots such as shown in the right panel normalised to 12 µM ATP conditions. Data are shown as mean ± SD (n=2), biologically independent experiments. *p<0.03, as determined by one-way ANOVA with Dunnett's multiple comparison test. Source data are provided as a Source Data file.

References:

1. Axten, J. M. *et al.* Discovery of GSK2656157: An Optimized PERK Inhibitor Selected for Preclinical Development. *Acs Med Chem Lett* 4, 964–968 (2013).
2. Fujimoto, J. *et al.* Identification of Novel, Potent, and Orally Available GCN2 Inhibitors with Type I Half Binding Mode. *Acs Med Chem Lett* 10, 1498–1503 (2019).
3. Madeira, F. *et al.* Search and sequence analysis tools services from EMBL-EBI in 2022. *Nucleic Acids Res* 50, W276–W279 (2022).
4. Murphy, J. M. *et al.* A robust methodology to subclassify pseudokinases based on their nucleotide-binding properties. *Biochem J* 457, 323–334 (2013).
