# Supplementary material for: Activation of the integrated stress response by inhibitors of its kinases
Source: Nat Commun. 2023 Sep 8;14:5535. doi: 10.1038/s41467-023-40823-8 (PMC10491595; doi:10.1038/s41467-023-40823-8)
Supplement: Supplementary file 3 — Reporting Summary [file 41467_2023_40823_MOESM3_ESM.pdf]

nature portfolio

Corresponding author(s): Anne Bertolotti

Last updated by author(s): Aug 8, 2023

Reporting Summary

Nature Portfolio wishes to improve the reproducibility of the work that we publish. This form provides structure for consistency and transparency in reporting. For further information on Nature Portfolio policies, see our Editorial Policies and the Editorial Policy Checklist.

Statistics

For all statistical analyses, confirm that the following items are present in the figure legend, table legend, main text, or Methods section.

n/a	Confirmed	
☐	☒	The exact sample size (n) for each experimental group/condition, given as a discrete number and unit of measurement
☐	☒	A statement on whether measurements were taken from distinct samples or whether the same sample was measured repeatedly
☐	☒	The statistical test(s) used AND whether they are one- or two-sided<br>*Only common tests should be described solely by name; describe more complex techniques in the Methods section.*
☐	☒	A description of all covariates tested
☐	☒	A description of any assumptions or corrections, such as tests of normality and adjustment for multiple comparisons
☐	☒	A full description of the statistical parameters including central tendency (e.g. means) or other basic estimates (e.g. regression coefficient) AND variation (e.g. standard deviation) or associated estimates of uncertainty (e.g. confidence intervals)
☐	☒	For null hypothesis testing, the test statistic (e.g. F, t, r) with confidence intervals, effect sizes, degrees of freedom and P value noted<br>*Give P values as exact values whenever suitable.*
☒	☐	For Bayesian analysis, information on the choice of priors and Markov chain Monte Carlo settings
☒	☐	For hierarchical and complex designs, identification of the appropriate level for tests and full reporting of outcomes
☒	☐	Estimates of effect sizes (e.g. Cohen's d, Pearson's r), indicating how they were calculated

Our web collection on statistics for biologists contains articles on many of the points above.

Software and code

Policy information about availability of computer code

Data collection	- Biorad ChemiDoc Touch system<br>- Licor Odyssey infrared imaging system<br>- GE Healthcare Typhoon Imager Scanner<br>- Wyatt Agilent 1200 series LC system with an online Dawn Helios ii system<br>- GE healthcare Akta with Superdex 200 Increase 10/300 GL column<br>- Prometheus NanoTemper<br>- Chimera X<br>- Clustal Omega
Data analysis	- Prism 9.4.1 (GraphPad Software, Inc)<br>- Licor ImageStudio Lite version 5.2.5

For manuscripts utilizing custom algorithms or software that are central to the research but not yet described in published literature, software must be made available to editors and reviewers. We strongly encourage code deposition in a community repository (e.g. GitHub). See the Nature Portfolio guidelines for submitting code & software for further information.

Data

Policy information about availability of data

All manuscripts must include a data availability statement. This statement should provide the following information, where applicable:

- Accession codes, unique identifiers, or web links for publicly available datasets
- A description of any restrictions on data availability
- For clinical datasets or third party data, please ensure that the statement adheres to our policy

Data generated or analysed during this study is now part of the source data file submitted together with the manuscript.

Human research participants

Policy information about studies involving human research participants and Sex and Gender in Research.

Reporting on sex and gender	No human participants were used in this study.
Population characteristics	n/a
Recruitment	n/a
Ethics oversight	n/a

Note that full information on the approval of the study protocol must also be provided in the manuscript.

Field-specific reporting

Please select the one below that is the best fit for your research. If you are not sure, read the appropriate sections before making your selection.

☒ Life sciences ☐ Behavioural & social sciences ☐ Ecological, evolutionary & environmental sciences

For a reference copy of the document with all sections, see nature.com/documents/nr-reporting-summary-flat.pdf

Life sciences study design

All studies must disclose on these points even when the disclosure is negative.

Sample size	Sample size for each experiment was determined based on previous studies (Tsaytler et al 2011, Das et al 2015, Krzyzosiak et al 2018, Schneider et al 2020, Pitera et al 2022).
Data exclusions	No data was excluded from analysis.
Replication	To ensure reproducibility of the results, the experiments have been performed as multiple biologically independent experiments, the exact number of n numbers is provided in the legends of the corresponding figure. Additionally, where possible more than one readout has been analysed to support the conclusions.
Randomization	For in vivo experiments the animals were randomly divided into the experimental groups by the animal facility staff. For other experiments samples were randomly allocated into groups.
Blinding	For in vivo experiments the animal technician performing the experiments was blinded. The responsible scientist that analyzed the data was not blinded.

Reporting for specific materials, systems and methods

We require information from authors about some types of materials, experimental systems and methods used in many studies. Here, indicate whether each material, system or method listed is relevant to your study. If you are not sure if a list item applies to your research, read the appropriate section before selecting a response.

Materials & experimental systems

n/a	Involved in the study	
☐	☒	Antibodies
☐	☒	Eukaryotic cell lines
☒	☐	Palaeontology and archaeology
☐	☒	Animals and other organisms
☒	☐	Clinical data
☒	☐	Dual use research of concern

Methods

n/a	Involved in the study	
☒	☐	ChIP-seq
☒	☐	Flow cytometry
☒	☐	MRI-based neuroimaging

Antibodies

Antibodies used

1. α-tubulin (Sigma Aldrich, #T5168, clone: B-5-1-2, 1:5,000) RRID:AB_477579
2. p-eIF2α (Abcam, #ab32157, clone: E90, 1:1,000) RRID:AB_732117
3. eIF2α (Abcam, #ab26197, 1:1,000) RRID:AB_2096478
4. eIF2α (Cell Signaling, #L57A5, clone: 2103, 1:1,000) RRID:AB_836874
5. PERK (Cell Signaling, #C33E10, clone: 3192, 1:1,000) RRID:AB_2095847
6. PERK (Cell Signaling, #D11A8, clone: 5683, 1:1,000) RRID:AB_10841299
7. ATF4 (Proteintech, #10835-1-AP, 1:1,000) RRID:AB_2058600
8. GCN2 (Cell Signaling, #E9H6C, clone: 40457, 1:1,000) RRID:AB_2799177
9. GCN2 (Cell Signaling, #3302S, 1:1,000) RRID:AB_2277617
10. p-GCN2 (Abcam, #ab75836, clone: EPR2320Y, 1:1,000) RRID:AB_1310260
11. PKR (Santa Cruz, #B-10, clone: sc-374015, 1:1,000) RRID:AB_628150
12. Vinculin (Cell Signaling, #4650S, 1:1,000) RRID:AB_10559207
13. anti-mouse (Promega, #W402B, 1:5,000) RRID:AB_430834
14. anti-rabbit (Promega, #W401B, 1:10,000) RRID:AB_430833
15. Goat anti-Mouse Alexa Fluor 680 (Invitrogen, #A32729, 1:5,000) RRID:AB_2633278
16. Goat anti-Rabbit Alexa Fluor 790 (Invitrogen, #A27041, 1:10,000) RRID:AB_2536102

Validation

1. https://www.sigmaaldrich.com/GB/en/product/sigma/t5168?gclid=Cj0KCQiAvqGcBhCJARIsAFQ5ke6ilqON_-ZXkfxLis8w1pXMFj1Lh4D2-KLrRsgQMrG-Q15G9c8zfDoaAusEEALw_wcB&gclsrc=aw.ds
2. https://www.abcam.com/eif2s1-phospho-s51-antibody-e90-ab32157.html
3. https://www.abcam.com/eif2s1-antibody-ab26197.html
4. https://www.cellsignal.com/products/primary-antibodies/eif2a-l57a5-mouse-mab/2103
5. https://www.cellsignal.com/products/primary-antibodies/perk-c33e10-rabbit-mab/3192
6. https://www.cellsignal.com/products/primary-antibodies/perk-d11a8-rabbit-mab/5683
7. https://www.ptglab.com/products/ATF4-Antibody-10835-1-AP.html
8. https://www.cellsignal.com/products/primary-antibodies/gcn2-e9h6c-rabbit-mab/40457
9. https://www.cellsignal.com/products/primary-antibodies/gcn2-antibody/3302
10. https://www.abcam.com/gcn2-phospho-t899-antibody-epr2320y-ab75836.html
11. https://www.scbt.com/p/pkr-antibody-b-10
12. https://www.cellsignal.com/products/primary-antibodies/vinculin-antibody/4650
13. https://www.promega.co.uk/products/protein-detection/primary-and-secondary-antibodies/anti_mouse-igg-h-and-l-hrp-conjugate/?catNum=W4021
14. https://www.promega.co.uk/products/protein-detection/primary-and-secondary-antibodies/anti-rabbit-igg-h-and-l-hrp-conjugate/?catNum=W4011
15. https://www.thermofisher.com/antibody/product/Goat-anti-Mouse-IgG-H-L-Highly-Cross-Adsorbed-Secondary-Antibody-Polyclonal/A32729
16. https://www.thermofisher.com/antibody/product/Goat-anti-Rabbit-IgG-Heavy-chain-Secondary-Antibody-Recombinant-Polyclonal/A27041

Eukaryotic cell lines

Policy information about cell lines and Sex and Gender in Research

Cell line source(s)	HeLa cells, RRID:CVCL_0030, Sigma-Aldrich IGBMC, Illkirch, France<br>SF9 cells, RRID:CVCL_0549, ThermoFisher Scientific, Cat#11496015
Authentication	The cell lines were authenticated by morphology.
Mycoplasma contamination	The frozen stocks of cell lines were mycoplasma free. The cell lines were not retested for mycoplasma contamination during the course of this study.
Commonly misidentified lines<br>(See ICLAC register)	None

Animals and other research organisms

Policy information about studies involving animals; ARRIVE guidelines recommended for reporting animal research, and Sex and Gender in Research

Laboratory animals	Mice: C57BL/6J RRID:IMSR_JAX:000664 males from 11 to 13 weeks old were used in the study.
Wild animals	No wild animals were used in the study.
Reporting on sex	The experiments have been performed on male mice to facilitate the experimental design. No conclusions regarding gender influence on the detected cell signalling changes are taken.
Field-collected samples	No field collected samples were used in the study.
Ethics oversight	All animal care and procedures were performed in compliance with the regulation on the use of Animals in Research (UK Animals Scientific Procedures Act of 1986 and the EU Directive 2010/63/EU) under the project license number P9DCDB3B0 and with approval from the LMB Animal Welfare and Ethical Review committee.

Note that full information on the approval of the study protocol must also be provided in the manuscript.
